# Polyploidy versus endosymbionts in obligately thelytokous thrips

**DOI:** 10.1186/s12862-015-0304-6

**Published:** 2015-02-22

**Authors:** Duong T Nguyen, Robert N Spooner-Hart, Markus Riegler

**Affiliations:** Hawkesbury Institute for the Environment, University of Western Sydney, Locked Bag 1797, Penrith, NSW 2751 Australia; School of Science and Health, University of Western Sydney, Locked Bag 1797, Penrith, NSW 2751 Australia

**Keywords:** Haplodiploidy, Thelytoky, Heterozygosity, Polyploidy, Hexaploidy, *Heliothrips haemorrhoidalis*, Thysanoptera, *Wolbachia*, *histone 3*

## Abstract

**Background:**

Thelytoky, the parthenogenetic development of females, has independently evolved in several insect orders yet the study of its mechanisms has so far mostly focussed on haplodiploid Hymenoptera, while alternative mechanisms of thelytoky such as polyploidy are far less understood. In haplodiploid insects, thelytoky can be encoded in their genomes, or induced by maternally inherited bacteria such as *Wolbachia* or *Cardinium*. Microbially facilitated thelytoky usually results in complete homozygosity due to gamete duplication and can be reverted into arrhenotoky, the parthenogenetic development of males, through treatment with antibiotics. In contrast, genetically encoded thelytoky cannot be removed and may result in conservation of heterozygosity due to gamete fusion. We have probed the obligate thelytoky of the greenhouse thrips, *Heliothrips haemorrhoidalis* (Bouché), a significant cosmopolitan pest and a model species of thelytoky in the haplodiploid insect order Thysanoptera. Earlier studies suggested terminal fusion as a mechanism for thelytoky in this species, while another study reported presence of *Wolbachia*; later it was speculated that *Wolbachia* plays a role in this thrips’ thelytokous reproduction.

**Results:**

By using PCR and sequence analysis, we demonstrated that global population samples of *H. haemorrhoidalis* were not infected with *Wolbachia, Cardinium* or any other known bacterial reproductive manipulators. Antibiotic treatment of this thrips did also not result in male production. Some individuals carried two different alleles in two nuclear loci, *histone 3* and *elongation factor 1 alpha*, suggesting heterozygosity. However, the majority of individuals had three different alleles suggesting that they were polyploid. Genetic diversity across both nuclear loci was low in all populations, and absent from mitochondrial *cytochrome oxidase I*, indicating that this species had experienced genetic bottlenecks, perhaps due to its invasion biology or a switch to thelytoky.

**Conclusions:**

Geographically broad sampling and experimental manipulation revealed low genetic diversity, absence of *Wolbachia* but presence of three different alleles of nuclear loci in most analysed individuals of obligately thelytokous *H. haemorrhoidalis*. This suggests that polyploidy may be involved in the thelytokous reproduction of this thrips species, and polyploidy may be a contributing factor in the reproduction of Thysanoptera and other haplodiploid insect orders.

**Electronic supplementary material:**

The online version of this article (doi:10.1186/s12862-015-0304-6) contains supplementary material, which is available to authorized users.

## Background

Thelytoky, or the parthenogenetic development of females from unfertilised eggs, has mostly been studied in species of insect orders with haplodiploid sex determination systems such as Hymenoptera [1]. Most haplodiploid species display arrhenotoky, where unfertilised eggs develop into haploid males and fertilised eggs into diploid females; in thelytoky diploid females develop from unfertilised eggs [1,2]. In a number of insect orders thelytoky can also be linked with polyploidy; however, polyploidy is considered rare, with most reports from within the order Coleoptera [3].

Thelytoky can either be obligate or facultative [4]; males are generally absent in obligately thelytokous populations, with the exception of the rare production of males [1]. Apomixis is thelytoky that occurs without meiosis; it results in offspring that are identical copies of the maternal genetic makeup. In contrast, automixis includes meiosis with one of at least three different avenues of diploidy restoration [1,2]. In gamete duplication, the diploidisation is accomplished by the duplication of the haploid genome of single nuclei after the second meiotic division, and hence results in homozygosity. In terminal fusion, the egg nucleus and second polar body fuse after the second meiotic division to give rise to the embryo, while in central fusion two polar bodies fuse to form the cell from which the embryo is derived [4]. Central fusion can maintain or progressively erode heterozygosity, with a transition rate of a heterozygous locus to homozygosity of 0 (without recombination) or 1/3 (with recombination and at loci distant enough from the centromere), respectively. In contrast, terminal fusion enforces homozygosity, with a transition rate of a heterozygous locus to homozygosity of 1 (without recombination) or 1/3 (with recombination and loci distant enough from the centromere) [1,5].

Thelytokous parthenogenesis can be encoded in arthropod genomes or can be induced by inherited microbial endosymbionts. The majority of endosymbiont*-*induced thelytoky is automixis with gamete duplication [1,6], except for bacterially facilitated apomictic thelytoky that was recorded in *Rickettsia*-infected *Neochrysocharis formosa* parasitoids [7] and *Wolbachia*-infected phytophagous *Bryobia praetiosa* mites [8]. The most common endosymbiont that induces thelytoky is *Wolbachia,* a ubiquitous maternally inherited intracellular bacterium of arthropods [9]. *Wolbachia* was first found to induce thelytoky in *Trichogramma* wasps [10]. After removal by antibiotic treatment and exposure to high temperatures, thelytoky was converted into arrhenotoky [10]. Subsequently, *Wolbachia* was confirmed to induce parthenogenesis in mites, springtails and a number of insect orders, in particular in Hymenoptera [6,11]. In Thysanoptera, *Wolbachia* was also found to cause thelytokous parthenogenesis in *Hercinothrips femoralis* [12] and *Franklinothrips vespiformis* [13]. In addition to *Wolbachia*, other bacterial endosymbionts including *Cardinium* [14-18] and *Rickettsia* [7,19-21] also cause thelytoky in arthropods other than thrips.

Thysanoptera is an insect order of over 6,000 recognised species [22] in which haplodiploidy has independently evolved [23,24]. Thelytoky occurs in a number of thrips species, however, it is far less studied in this order than in Hymenoptera. The greenhouse thrips, *Heliothrips haemorrhoidalis* (Bouché) (Thysanoptera: Thripidae), an economically important pest of numerous crops worldwide, is obligately thelytokous [25]. Males are absent from laboratory and most field populations. Yet, exceptional males have been found, mostly in South America [26,27]. The incidence of males in South America, especially Brazil and Peru, plus the discovery of two closely related species *Heliothrips sylvanus* and *Heliothrips zucchi* in this biogeographic region indicates that *H. haemorrhoidalis* is native to the Amazon basin [26].

*Heliothrips haemorrhoidalis* is one of the first thrips species for which cytological mechanisms of thelytoky have been studied [28]. Bournier [29] described the thelytokous parthenogenesis in *H. haemorrhoidalis* as automictic parthenogenesis; meiosis was observed as the chromosome number was reduced from 42 to 21 during oocyte formation, followed by the re-establishment of 42 chromosomes through fusion of the second polar body with the egg nucleus. According to these results, Bell [30] and Suomalainen *et al.* [4] concluded that thelytoky in *H. haemorrhoidalis* was automixis through terminal fusion. Later, this species tested positive for *Wolbachia* by PCR using *Wolbachia* specific primers for the cell cycle *ftsZ* gene [31]. However, these positive PCR products were not sequenced nor confirmed, and the potential role of *Wolbachia* in the thelytokous reproduction of this species was not tested further through antibiotic treatment. In the absence of adequate confirmation of these previous results, a number of papers suggested a role of *Wolbachia* in thelytoky of this thrips species [11,12,32-34].

In our study, we tested thelytokous *H. haemorrhoidalis* collected from Africa, Asia, Australasia, Europe and South America for *Wolbachia* and other potential parthenogenesis-inducing bacterial endosymbionts such as *Cardinium* by using PCR and DNA sequence analysis. We also treated one Australian *H. haemorrhoidalis* laboratory population with antibiotics in order to test whether thelytokous reproduction of this species could be reverted to arrhenotoky. We then analysed the genetic and allelic diversity of individuals in one mitochondrial and two nuclear loci, in particular to assess whether individuals are homozygous, heterozygous or polyploid. We hypothesised that homozygosity in nuclear loci would indicate gamete duplication (as commonly seen in endosymbiont-facilitated thelytoky) or terminal fusion, while heterozygosity could indicate central fusion. More than two nuclear alleles per individual would indicate polyploidy. We also aimed to characterise the genetic identity and diversity of cosmopolitan populations as limited genetic information had been available for this invasive thrips species prior to our study.

## Methods

### Thrips collection and cultures

Our study included specimens of six *H. haemorrhoidalis* populations that were field sampled from Australia, Chile, Japan, South Africa, Spain and United Kingdom (Additional file 1: Table S1) and from three laboratory populations, established from individuals collected in Richmond (New South Wales, Australia) in 1995 [35], Canberra (Australian Capital Territory) in 2011, and Te Puke (New Zealand) in 2013. The two Australian laboratory populations were maintained on citrus fruits [36]. Individuals were reared in plastic boxes (25 × 17 × 20 cm) with a lid that had a thrips-proof mesh (mesh aperture 160 × 160 μm). About 15–20 insecticide-free large, green and unripe oranges (*Citrus × aurantium* L. ‘Washington navel’) harvested from orchards on the UWS Hawkesbury campus were washed under tap water to remove any insects, dried with paper towel and placed tightly in each box together with approximately 100 thrips individuals. Each generation was left undisturbed for three weeks. Every three weeks, old and deteriorated fruits were removed and replaced with fresh fruits. The two Australian laboratory populations were reared in separate boxes that were kept at room conditions (22 ± 2°C) in different laboratories in order to avoid cross contamination. Morphological characters of thrips specimens were examined to confirm their identity, following Mound and Monteiro [27] and Mound *et al.* [37].

As sequences of the analysed *elongation factor 1α* (*EF1a*) gene region from other thrips species were not available on GenBank, we also included individuals of *Frankliniella occidentalis*, *Pezothrips kellyanus*, *Thrips tabaci* and *Thrips imaginis* (Additional file 1: Table S1).

### DNA extraction

For the characterization of mitochondrial and nuclear markers we analysed individuals while the screening for *Wolbachia* included extracts from both, individuals and pooled individuals. DNA was extracted from individual adults, or pools of five, ten, or 20 larvae, pupae or adults using GenElute™ Mammalian Genomic DNA Miniprep Kit (Sigma Aldrich, Missouri, USA). Extraction steps were undertaken following the protocols of the manufacturer, apart from the DNA elution step, where all samples were eluted with 50 μL elution solution. Prior to DNA extraction, the sample preparation process included surface sterilization, using the methods described by Hail *et al.* [38] to avoid contamination with environmental bacteria. First, samples were dipped in absolute ethanol for 1 min, then in sodium hypochlorite solution (reagent grade, available chlorine 10-15%; Sigma-Aldrich) for 5 min. Finally, the samples were rinsed in ultrapure-filtered distilled water for 1-2 min. The quality of extracted DNA was assessed through PCR amplifications of the mitochondrial cytochrome *c* oxidase subunit I (*COI*) gene.

### Screening of thrips for wolbachia and cardinium

A range of *Wolbachia* specific primer pairs for *wsp* [39]*,* 16S rDNA [40,41] and five loci of the multilocus sequence typing (MLST) system [42]: *ftsZ* [43,44], *gatB*, *coxA, hcpA* and *fpbA* (Additional file 2: Table S2) were used to screen individuals for *Wolbachia* infections. In addition, we also tested primers and PCR conditions that had previously been evaluated by Simões *et al.* [45] to detect different *Wolbachia* strains in a wide range of hosts. Based on their results, we used the most sensitive primer pairs 553F_W and 1334R_W, WspecF and WspecR, and fpbA F1 and R1. To maximise detection of *Wolbachia* present at low titre [46], long PCR [47], and nested and semi-nested *Wolbachia-*specific PCR approaches targeting 16S rDNA*, wsp* and MLST loci (Additional file 3: Table S3) were also performed on Australian field and laboratory individuals. Extracts of *Drosophila simulans* (Riverside) infected with *Wolbachia* strain *w*Ri and *Drosophila melanogaster* (W1118) infected with *w*MelPop were used as positive controls, while negative controls were tetracycline treated *D. melanogaster* (CST) as well as no-template controls. Samples were also screened for *Cardinium* using the primer pairs ChF and ChR [15,17], and CLOf1 and CLOr1 [48] (Additional file 2: Table S2). *Encarsia perniciosi* was used as positive control for *Cardinium* specific PCR assays [49]. PCR protocols were as listed in Additional file 4: Table S4 and Additional file 5: Table S5. PCR products were run in agarose gels (1%) stained with ethidium bromide at 90 V for 40 min and visualised by UV transillumination.

### Characterisation of other bacterial symbionts

In order to detect other possible bacterial symbionts, the primer pairs 61F and 1227R, and 10F and 1507R [41] were used to amplify eubacterial 16S rDNA in individuals from the Chilean and two Australian (Richmond, Canberra) populations (Additional file 3: Table S3). Products obtained from these PCR reactions were cloned and sequenced. Column purified PCR products were ligated into pGEM-T Easy Vector (Promega) followed by transformation of JM109 High Efficiency Competent Cells (Promega) according to the manufacturer’s protocol. White colonies containing inserts were picked, transferred into 20 μl colourless 5× GoTaq reaction buffer (Promega), boiled at 98°C for 10 min, and 1 μl was used as template in colony PCR reactions using the SP6 and T7 promoter primers [50]. Colony PCR products were treated with 2 μl of ExoSAP mixture containing 0.5u Exonuclease I (New England Biolabs, Ipswich, MA) and 0.25u Shrimp Alkaline Phosphatase (Promega), then incubated at 37°C for 30 min, followed by 95°C for 5 min [50]. The PCR products were then sent to Macrogen Korea for sequencing. PCR conditions, the number of sequenced clones, GenBank accession numbers and related information are included in Additional file 4: Table S4, Additional file 5: Table S5, Additional file 6: Table S6 and Additional file 7: Table S7.

### Antibiotic treatments

Two experiments were undertaken in order to test whether thelytoky could be removed from *H. haemorrhoidalis* by antibiotic treatment. A first experiment established the response of adult thrips to two commonly used antibiotics, rifampicin and tetracycline hydrochloride at three concentrations (w/v): 0.5%, 2.5% and 5%. Antibiotic powder (Sigma-Aldrich) was mixed with 50% w/v *Apis mellifera* honey and distilled water; absorbent cotton wool soaked with this mixture was provided to newly emerged adult thrips for seven days. Thrips in the control treatment were provided with cotton wool soaked with honey-water solution only. Neutral red added to the antibiotic solution confirmed that the thrips drank the antibiotic solution, as after two days the thrips’ digestive tracts were coloured red. After treatment, ten survivors from each treatment were transferred as a cohort to clean Petri dishes (14 cm diameter) containing a Washington navel orange leaf as a food source and for oviposition. Mature leaves were harvested for the experiment to support oviposition by *H. haemorrhoidalis* based on its habit not to oviposit in fresh young leaves [51]. Harvested leaves were cleaned under tap water and dried with a paper towel. The leaves were kept fresh for several days by wrapping the petiole in absorbent cotton wool soaked with water. Every seven days, the thrips were transferred to new Petri dishes with a fresh leaf. Leaves from each week containing eggs were incubated separately to allow hatching of larvae. The number of offspring of treated females over a three-week period was recorded and all offspring were checked for presence of any males.

In a second experiment, both larvae and adults were treated with rifampicin (5% w/v) in order to extend the exposure time of thrips to antibiotics. Cotton wool soaked with rifampicin solution was provided to second instar *H. haemorrhoidalis* larvae for five days prior to their pupation. Adult thrips that developed from these treated larvae were fed on this solution for another five days. The soaked cotton wool was replaced with freshly prepared rifampicin honey-water solution every two days. Thrips in the control treatment were offered cotton wool soaked with honey-water without antibiotics. After treatment, 25 survivors were transferred individually to clean Petri dishes (14 cm diameter) containing an orange leaf as previously described. Every seven days, females were individually transferred to new Petri dishes with a fresh leaf. The leaves containing eggs from individual females were kept separately to rear their offspring. Eggs were collected over a three-week period. The number of offspring of individual females was recorded and the offspring were checked for presence of males. The experiment was conducted in an incubator at 25 ± 2°C, 70% relative humidity and 16:8 light/dark photoperiod. The numbers of offspring produced by females within the first, second and third week after treatment were compared using Independent-Samples *T* test in SPSS 21 (IBM Corporation, Armonk, NY, USA).

### Sequencing of mitochondrial and nuclear DNA markers

A segment of the mitochondrial *COI* was amplified by PCR using the primer pair LCO1490 and HCO2198 [52]. PCR conditions were as described by Rugman-Jones *et al.* [53] (Additional file 4: Table S4 and Additional file 5: Table S5). PCR products obtained from three individual thrips collected from each of nine populations (Additional file 1: Table S1) were treated with the ExoSAP mixture and direct sequenced as described above.

Two nuclear gene fragments of *H. haemorrhoidalis*, histone *H3* (~450 bp) and *EF1a* (~860 bp), were amplified. For *H3*, primers H3AF and H3AR were used [54]; and for *EF1a of H. haemorrhoidalis*, primers were modified from EF1aF [55] and rcM4 [56] while EF1aF and rcM4 were used to amplify this locus from all other thrips species. In order to investigate the heterozygosity and geographic variation in these loci, one individual thrips from each of eight *H. haemorrhoidalis* populations was selected (Additional file 1: Table S1). PCR conditions were as described in Additional file 4: Table S4 and Additional file 5: Table S5. Direct sequencing of the two nuclear genes resulted in ambiguous target sequences with multiple peaks. For this reason, the amplified PCR products were cloned prior to sequencing following the procedures described above. Colony PCR amplicons were treated with ExoSAP and submitted to sequencing by using the listed plasmid primers. For each individual, three to six clones were sequenced in order to obtain the individual’s allelic sequences.

### Phylogenetic and sequence analyses

DNA sequences were assembled by Sequencher v4.10.1 (Gene Codes Corporation, Michigan, USA) and the plasmid vector and primer sequences removed. Mitochondrial and nuclear protein-coding sequences were checked for open reading frames, stop codons or unexpected indels; this was to ensure that the target gene had been sequenced [57]. For *COI*, BLAST (NCBI, Maryland, USA) was employed to identify similar sequences from GenBank which were then included in the alignment calculated with Muscle in MEGA 5.2.2 [58]. Before constructing a phylogenetic tree, the reliability of alignments was checked by determining the average p-distances (<0.8) of the protein alignments [59]. The best evolution model for phylogenetic analyses was also tested, and phylogenetic trees of the entire sequence were constructed by Bayesian Inference using MrBayes v3.2.2 [60]. For *H3* and *EF1a*, the intron splicing junctions were identified and compared to other insect species. It had been previously reported that cloning experiments would reveal PCR *Taq* DNA polymerase errors at the rate of 1:1000 bases [61], although the GoTaq DNA polymerase (Promega) used in this experiment had a lower error rate [62]. In order to control for *Taq* DNA polymerase errors, we followed the approach for removing singletons described by Villablance *et al.* [61]. Finally, sequences were aligned with Muscle in MEGA 5.2.2 in order to identify the polymorphic sites. GenBank accession numbers of *COI, H3* and *EF1a* sequences were represented in Additional file 7: Table S7.

## Results

### *Analysis of bacterial symbionts of* H. haemorrhoidalis

This study looked at 638 specimens from nine populations, including three laboratory populations from Australia and New Zealand, and six field populations from five different continents (Table 1). All specimens were sexed, and none was male. By using standard PCR on 16S rDNA*, ftsZ*, *wsp* and all MLST loci, *Wolbachia* was not detected in any DNA extract of 45 field specimens collected from Australia, Chile, Japan, South Africa, Spain and United Kingdom. Specimens of a New Zealand laboratory population (n = 10) were also negative for *Wolbachia*, and so were all 85 and 28 DNA extracts involving 543 and 40 Australian laboratory individuals from Richmond and Canberra, respectively, including all extracts from pooled specimens (Additional file 8: Table S8). Long PCR and nested PCR also revealed absence of infection in all Australian samples*.* Furthermore, we tested almost all *Wolbachia* primers that had previously been used for screening projects (Additional file 2: Table S2 and Additional file 3: Table S3), including the primer pair ftsZ102 FOR - ftsZ969 REV designed by Holden *et al.* [43] which was used for *Wolbachia* detection in *H. haemorrhoidalis* in the study by Pintureau *et al.* [31]. With this primer pair we obtained a single faint band at 55°C annealing temperature and a double band at 50°C annealing temperature for only one DNA extract of 10 pooled adult specimens from Richmond. These amplicons were excised from the gel, purified and cloned. Sequencing of cloned fragments did not result in *Wolbachia* sequences, so we concluded that those positive bands were artefacts produced by *ftsZ* primers on *H. haemorrhoidalis* extracts. In contrast, samples of positive controls, *Drosophila simulans* (Riverside) and *Drosophila melanogaster* (W1118) in all PCR assays resulted in strong positive bands at the expected size of *Wolbachia*.

**Table 1:** **Number of DNA extracts of laboratory and field individuals tested for** ***Cardinium*** **and** ***Wolbachia***

**#**	**Location**	***Cardinium***		***Wolbachia***	
		**n extracts**	**n individuals**	**n extracts**	**n individuals**
**Laboratory populations**		**32**	**50**	**123**	**593**
1	Australia, NSW	16	34	85	543
2	Australia, ACT	6	6	28	40
3	New Zealand	10	10	10	10
**Field populations**		**45**	**45**	**45**	**45**
1	Australia, QLD	3	3	3	3
2	South Africa	10	10	10	10
3	Japan	6	6	6	6
4	Spain	10	10	10	10
5	United Kingdom	6	6	6	6
6	Chile	10	10	10	10
**Total**		**77**	**95**	**168**	**638**

Subtotals of individuals from laboratory and field populations are in bold. Australian individuals were from Richmond (New South Wales NSW), Canberra (Australian Capital Territory ACT) and Sunshine Beach (Queensland QLD).

Field (n = 45) and laboratory (n = 32) specimens, including individual and pooled samples of different life stages (Additional file 8: Table S8), were also screened for *Cardinium* 16S rDNA; all 77 *H. haemorrhoidalis* extracts were negative (Table 1) while all *E. perniciosi* controls were positive. In order to characterise bacteria present in *H. haemorrhoidalis*, we applied the universal eubacterial primer pairs 61F-1227R and 10F-1507R [41] for PCR and isolated sequences through cloning experiments. BLAST analysis revealed that 4 out of 19 cloned sequences from 8 individuals (including larval and adult stages) had a 99% match with *Citrus sinensis* chloroplast (Genbank accession number DQ864733). Other sequences indicated the presence of bacteria closely related to Enterobacteriaceae previously described as gut symbionts from thrips and other insects (Additional file 6: Table S6). The analysis of eubacteria 16S rDNA did not reveal the presence of *Wolbachia, Cardinium* or any other known intracellular bacteria.

### Antibiotic treatment

Neither antibiotic treatment experiments resulted in the production of male offspring (Table 2 and Additional file 9: Figure S1). Tetracycline treatment of adults appeared to result in higher mortality and lower fecundity than treatment with rifampicin. Rifampicin was subsequently used to treat second instars and adult stages; this also significantly reduced lifetime fecundity of treated thrips. Within the first week of oviposition after treatment, thrips produced significantly fewer eggs than control thrips (*t* = 2.76, *df* = 48, *p* = 0.008) (Table 2). This negative impact of rifampicin was still detected in the second week (*t* = 2.42, *df* = 48, *p* = 0.02), but was not significant in the third week of egg-laying (*t* = 1.93, *df* = 48, *p* = 0.06). Overall, antibiotic treated females produced significantly fewer offspring in the three weeks following treatment than untreated females (*t* = 4.26, *df* = 48, *p* < 0.0001).

**Table 2:** **Sex ratio and mean offspring number of** ***Heliothrips haemorrhoidalis***

**Treatment**	**1st week**		**2nd week**		**3rd week**		**Total progeny in 3 weeks**	
	**Female**	**Male**	**Female**	**Male**	**Female**	**Male**	**Female**	**Male**
Rifampicin (5%)	2.08 ± 0.23	0	8.56 ± 0.45	0	11.52 ± 0.46	0	22.16 ± 0.52	0
Control	3.04 ± 0.26	0	10.04 ± 0.41	0	12.84 ± 0.50	0	25.92 ± 0.70	0
*t*-test (df 48)	2.76		2.42		1.93		4.26	
*p*	0.008		0.02		0.06		<0.0001	

This was tested on 25 females during the first, second and third week after rifampicin treatment (both larval and adult stages; second experiment). Differences between treatment and control were tested with an Independent-Samples *T* test.

### *Genetic characterisation of* H. haemorrhoidalis

Sequencing of *COI* of 27 individuals collected from different continents resulted in 680 bp fragments without any polymorphic sites. This sequence was identical to the *H. haemorrhoidalis COI* (GenBank accession number KC513162) collected from ACT, Australia [54]. The sequences contained no stop codon or indel. Phylogenetic analysis together with other thrips sequences from GenBank based on Bayesian Inference, model GTR + G + I showed that *H. haemorrhoidalis* sits within the subfamily Panchaetothripinae (Additional file 10: Figure S2).

PCR amplifications of *H3* gene fragments of *H. haemorrhoidalis* samples collected from different regions resulted in a strong amplicon of approximately 450 bp. Direct sequencing of these fragments produced ambiguous sequences with mixed peaks. Therefore, PCR products from eight individuals, one each from eight populations, were cloned. This resulted in 33 sequenced clones with a length of 439 bp, including a 109 bp intron (Additional file 11: Figure S3). In addition to this, a single clone of the Queensland individual had a 14 bp insertion within this intron region. There were no indels or stop codons within the 330 bp exon sequence of any individual.

**Figure 1:**
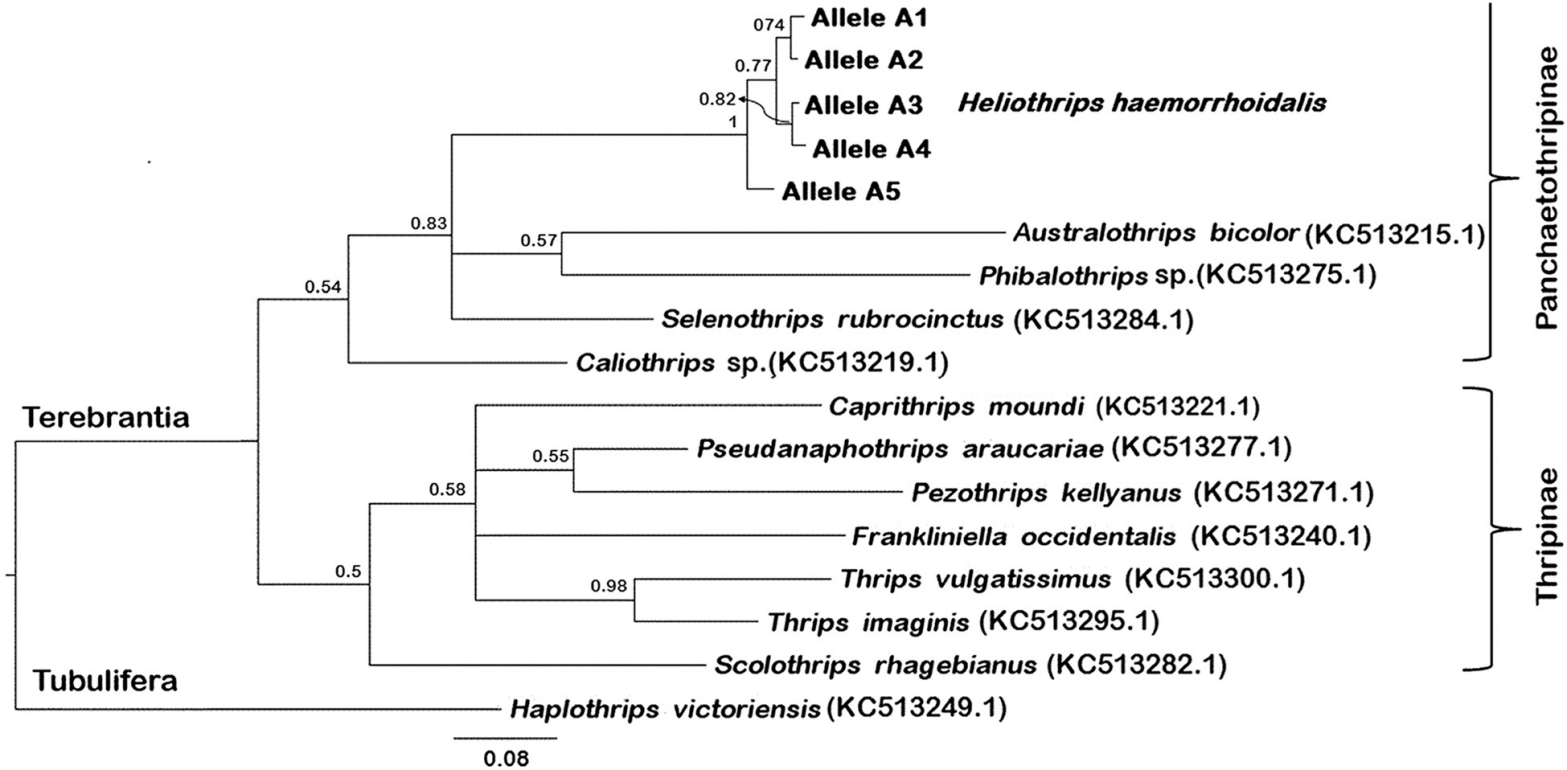
**Phylogenetic tree based on** ***H3*** **exon sequences** ***.*** This tree was constructed with five alleles of partial *H3* exons of *Heliothrips haemorrhoidalis* (318 bp) using Bayesian Inference (model T92 + G + I). The tree also includes other thrips species within the subfamilies Panchaetothripinae and Thripinae (both in the family Thripidae of the suborder Terebrantia). *Haplothrips victoriensis* (family Phlaeothripidae, suborder Tubulifera) was used as outgroup. Numbers at nodes represent posterior probabilities >50%. Scale bar represents the number of nucleotide substitutions per site.

Across the 33 cloned fragments with a 109 bp intron, three polymorphic sites were identified within the 330 bp exon sequence and no variation in the 109 bp intron (Additional file 11: Figure S3), while the single clone of Queensland had 20 polymorphic sites, mainly in the 123 bp intron region. Based on these polymorphic sites across these 34 cloned sequences, five *H3* alleles (A1-A5) were defined. All individuals had intra-individual diversity within this locus. Individuals from Australia (Canberra, ACT), New Zealand, Japan and United Kingdom contained two allelic variants, while individuals from Australia (Queensland), South Africa, Spain and Chile presented three *H3* alleles, suggesting that these individuals may be polyploid (Table 3, Additional file 11: Figure S3). Bearing in mind the small sample number, there was no unique difference between the individuals that could be indicative of geographic differentiation. Bayesian Inference analyses of *H3* sequences (without intron) confirmed the monophyletic position of the five *H3* alleles of *H. haemorrhoidalis* with the sequences of other thrips species within Panchaetothripinae subfamily, although, overall, the *H3* phylogeny is not well resolved (Figure 1).

**Table 3:** **Distribution of alleles across** ***Heliothrips haemorrhoidalis*** **individuals**

**a)**					
**#**	**Location**	***H3***		***EF1a***	
		**Alleles**	**Total**	**Alleles**	**Total**
1	Australia, ACT	A1, A2	2	A1, A2	2
2	Australia, Queensland	A1, A3, A5	3	A2, A3, A4	3
3	New Zealand	A1, A2	2	A2, A5	2
4	South Africa	A1, A3, A4	3	A2, A6, A7	3
5	Japan	A1, A4	2	A2, A5, A8	3
6	United Kingdom	A1, A4	2	A2, A5, A9	3
7	Spain	A1, A2, A4	3	A2	1
8	Chile 1	A1, A3, A4	3	A2, A6, A10	3
9	Chile 2	n.d.	n.d.	A2, A11, A12	3
**b)**					
	**Number of alleles**	**Number of individuals (** ***H3*** **)**		**Number of individuals (** ***EF1a)***	
	1 allele	0		1	
	2 alleles	4		2	
	3 alleles	4		6	

**a)** Number of *H3* and *EF1a* alleles (sequences included both introns and exons) detected in individuals of *H. haemorrhoidalis*; one individual per population was analysed (two Chilean individuals were analysed for *EF1a*); **b)** number of individuals for which one, two or three alleles of *H3* and *EF1a* were detected

ACT: Australian Capital Territory; n.d.: not determined.

Similarly to *H3*, direct sequencing of the *EF1a* gene resulted in ambiguous sequences with multiple peaks. Thus, we sequenced 42 clones from eight populations (one or two individuals each), and obtained 861 bp *EF1a* fragments containing two intron regions (Additional file 12: Figure S4). The exon region was 681 bp and did not reveal any stop codons or indels across individuals. The first intron was the same length (73 bp) for all individuals while the second intron of 41 cloned sequences included indels and ranged from 83 to 85 bp. We also found an additional cloned sequence (sequence Chile 8–1 in Additional file 12: Figure S4) in one individual from Chile that had a divergent nucleotide composition and insertion in the second intron (101 bp).

**Figure 2:**
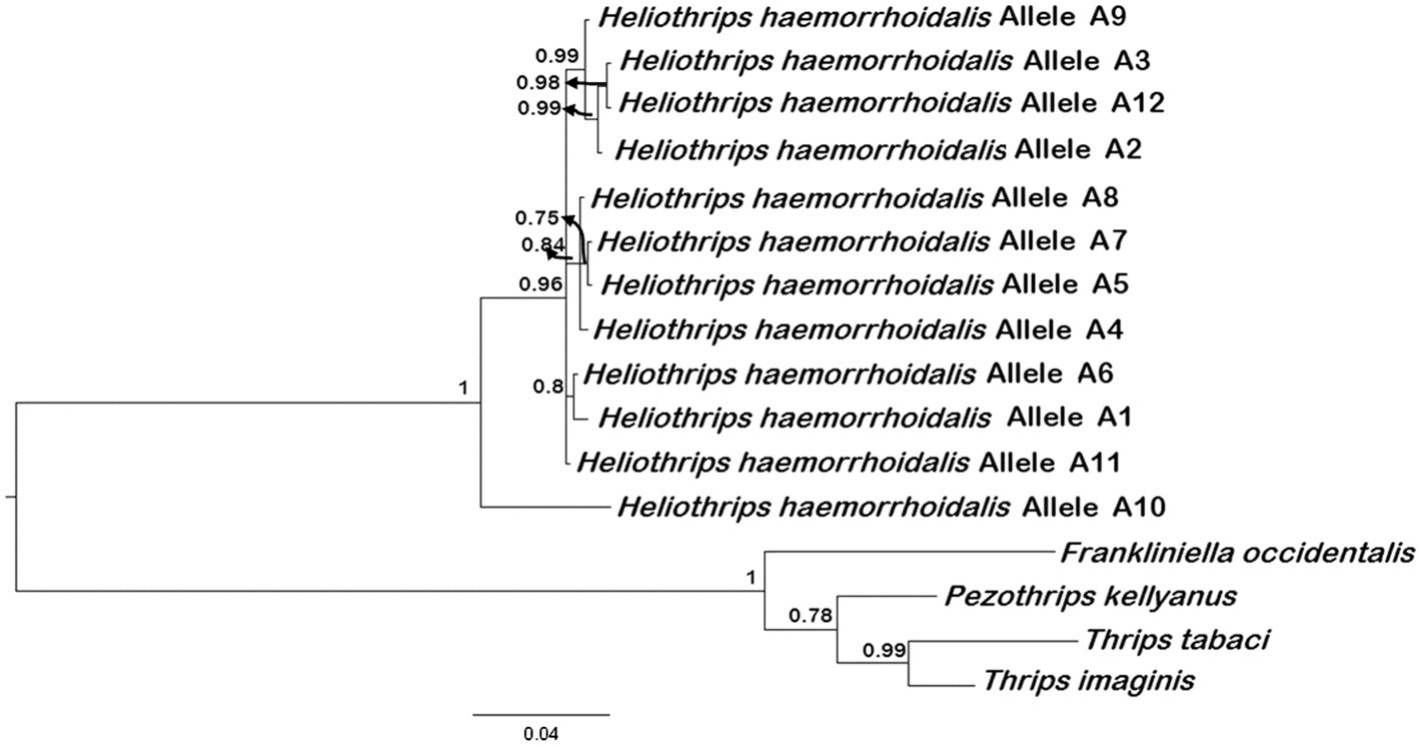
**Phylogenetic tree based on** ***EF1a*** **exon sequences.** The tree was constructed with 12 alleles of *Heliothrips haemorrhoidalis* (681 bp each) and other thrips species within the family of Thripidae using Bayesian Inference (model T92 + I). Numbers at nodes represent posterior probabilities >50%. Scale bar represents the number of nucleotide substitutions per site.

Across the 42 *EF1a* sequences, including introns, we identified 12 alleles (Table 3 and Additional file 12: Figure S4). Two to three alleles were found in all individuals, except for the individual from Spain, for which we sequenced nine clones; all nine were identical alleles, suggesting that this individual was homozygous in this locus (Additional file 12: Figure S4). Individuals from Canberra and New Zealand showed two allelic variants while others exhibited three allelic variants, suggesting that these individuals may be polyploid (Table 3 and Additional file 12: Figure S4). The distinct allele (Allele A10) that was detected in one Chilean individual was basal to the other alleles (Figure 2 and Additional file 13: Figure S5).

When looking at both nuclear loci, we found seven individuals that had three alleles in at least one of two loci, while two individuals (Australia ACT and New Zealand) had two alleles (Table 3).

Notwithstanding the limited number of individuals that were analysed for the two nuclear genes, the overall diversity of three genes *COI*, *H3* and *EF1a* in *H. haemorrhoidalis* collected from different countries was found to be low. The mean distance of 27 *COI* sequences across continents was nil and <1% for *H3* intron, *H3* exon and *EF1a* exon sequences, while it was 2.75% for the *EF1a* intron (Table 4).

**Table 4:** **Nucleotide distances**

**Locus**	**Overall mean distance (%)**	**Sequence length (bp)**
*COI*	0.0	680
*H3* exon	0.48	330
*H3* intron	0.70	123
*EF1a* exon	0.95	681
*EF1a* intron	2.75	180

Overall mean nucleotide distance across *COI, H3* and *EF1a* sequences of *Heliothrips haemorrhoidalis.*

## Discussion

In our study, all *H. haemorrhoidalis* population samples were all-female, and none were infected with *Wolbachia*, *Cardinium* or any other bacterial reproductive manipulators. Antibiotic treatment of a thelytokous laboratory population from Australia did not result in the production of males, indicating that thelytoky in *H. haemorrhoidalis* is not induced by symbiotic bacteria. Genetic characterisation of the two nuclear genes *H3* and *EF1a* revealed allelic diversity within individuals; unexpectedly most individuals had three allelic variants which could be indicative of polyploidy. No genetic diversity in mitochondrial *COI,* and low diversity in both nuclear genes *H3* and *EF1a,* suggest that this species may have experienced significant genetic bottlenecks possibly due to its invasion history, or due to an evolutionary switch from arrhenotokous to thelytokous reproduction.

### Thelytoky not caused by bacterial symbionts

All 638 tested field and laboratory individuals of *H. haemorrhoidalis* collected from seven countries of five different continents were negative for *Wolbachia* when tested with a large set of *Wolbachia* specific PCR primers. Highly sensitive PCR methods and testing of DNA samples extracted from single and pooled individuals of different developmental stages did also not provide any evidence for low-titre infections.

In contrast, an earlier study by Pintureau *et al.* [31] reported *Wolbachia* infection in *H. haemorrhoidalis* collected from southern France and southern Portugal based on PCR results from two DNA extracts of two to three pooled adults each, using the primers ftsZ102 FOR - ftsZ969 REV designed by Holden *et al.* [43]. It is possible that this was due to unspecific primer binding, artefact formation, contamination or thrips misidentification. We also obtained amplicons from one DNA extract when using the same *ftsZ* primers. However, after cloning and sequencing we confirmed that they were not *Wolbachia* sequences. Highly sensitive PCR assays can detect contaminations deriving from other species that interact with the target species [63]. Nault *et al.* [64] reported such an issue when testing *T. tabaci* for *Wolbachia*; however, negative results in repeated PCR assays using the same template DNA confirmed that the infection was the result of contamination. Pintureau *et al.* [31] also detected *Wolbachia* in *Megaphragma amalphitanum* (Hymenoptera: Trichogrammatidae)*,* an egg parasitoid of *H. haemorrhoidalis* and this parasitoid could have been the source of *Wolbachia* detection in *H. haemorrhoidalis*. Moreover, a confirmed *Wolbachia-*infected thrips species, the banded greenhouse thrips, *H. femoralis* [12], was involved in the same study and could have been another source of contamination or accidental misidentification. Unfortunately, no DNA sequence of this earlier *ftsZ* amplicon from *H. haemorrhoidalis* was reported to confirm and further characterise the *Wolbachia* strain. Despite this all, it is still possible that *H. haemorrhoidalis* tested by Pintureau *et al.* [31] was infected by *Wolbachia* as we did not assess populations from France and Portugal. However, in our study, all populations (including from Europe) tested negative.

Beyond this, all 77 samples tested for *Cardinium* were negative. Sequencing of the eubacterial 16S rDNA in *H. haemorrhoidalis* resulted in the detection of a number of insect gut symbionts previously described from thrips [65,66] but also of chloroplast DNA from the host plant, in both larval and adult stages. This may be indicative that thrips do not only ingest plant sap but cells containing chloroplasts, and chloroplast DNA specific assays of field specimens could be applied in order to characterise host plant associations of thrips species.

As we did not detect any known bacterial reproductive manipulators of insects in *H. haemorrhoidalis*, we concluded that its thelytoky is not induced by bacteria. This was subsequently confirmed by antibiotic treatment of a thelytokous laboratory population that did not result in arrhenotokous reproduction. Similarly, Nault *et al.* [64] and Kumm and Moritz [12] found that neither *Wolbachia* nor any other bacterial symbiont played a role in thelytokous *T. tabaci*, as antibiotic and heat treatments did not alter the sex ratio of this species. A recent study found *Wolbachia* at varying prevalence in the three thelytokous grass thrips species. However, *Wolbachia* appeared to not play a role in the thelytokous reproduction of *Aptinothrips karnyi* and *Aptinothrips stylifer* as antibiotic treatment did not remove thelytoky; removal of *Wolbachia* from thelytokous *Aptinothrips rufus,* however, resulted in production of some males [67].

### *Heterozygosity or polyploidy in* H. haemorrhoidalis*?*

In order to further investigate thelytoky in *H. haemorrhoidalis* we looked at intra-individual allelic diversity of *H3* and *EF1a* in eight and nine individuals, respectively, collected from eight different populations. Although, this is a low sample number for addressing more specific questions about population diversity, it is a large enough sample to investigate mechanisms of thelytoky in this species. Similar approaches have previously been used to clarify the type of diploidy restoration in thelytokous insects belonging to the orders Hymenoptera and Odonata [5,68,69].

Sequence analysis of *H3* revealed the unexpected presence of an intron in this gene while previous extensive analysis of *H3* of Thysanoptera using the same primers did not report any introns [54]. Based on its highly conserved amino acid sequence across all eukaryotes, *H3* can be separated into replication-coupled (RC, also known as H3.1 and H3.2) and replication-independent (RI or H3.3) variants [70]. All 33 cloned *H3* exons of *H. haemorrhoidalis* matched with the RC variant that is expected to not have introns [70]; all exon fragments appeared functional and were monophyletic, and the intron displayed common intron splice recognition sites. The nature of this unexpected *H3* intron may require further investigation.

The 843 bp *EF1a* sequence of *H. haemorrhoidalis* corresponded to position 598–1280 in the *EF1a* copy F1 of *D. melanogaster* [71,72], and contained two introns. The first intron was at the position 753/754, as seen in copy 1 of *EF1a* in Coleoptera, Dictyoptera, Hymenoptera, Hemiptera, Neuropterida and Odonata [71,72]. The second intron was in position 1150/1151, which had previously been found in three orders: Thysanoptera [73], Psocoptera and Hemiptera [71].

Within each individual we detected two to three allelic variants in both *H3* and *EF1a*, except for the Spanish individual which only had one allele of *EF1a,* while it had three allelic variants of *H3.* Two individuals had two alleles in both loci, suggesting heterozygosity, while seven individuals had three alleles in at least one of both nuclear loci. While it is possible that we isolated paralogous gene copies, it is less likely that this happened for both nuclear loci that have previously also been used for phylogenetic analyses [54]. Beyond this, we found the maximum number of alleles identified from any individual was three, which could indicate triploidy. Triploidy has previously been suggested for *H. haemorrhoidalis* by Bournier [29], so far the most authoritative text about chromosome numbers of this species; this would also fit its relatively higher chromosome number in comparison with other thrips species [74].

However, some of our findings are in conflict with assumptions about the detection of terminal fusion in this species [4,29,30], unless this was previously erroneously reported. Terminal fusion is expected to rapidly erode heterozygosity in individuals [1]. Furthermore, triploidy conflicts with automixis and meiotic division in general [75]. By analysing gametogenesis, Bournier [29] counted n = 21 chromosomes after reductional division, and 2n = 42 chromosomes after fusion. Earlier studies by Pomeyrol [28] and Pussard-Radulesco [76] suggested n = 16 and n = 26/28, respectively, however these studies were commented by Bournier [29] to be less accurate due to inadequate techniques used. A total chromosome number of 42 would allow either diploidy, triploidy or hexaploidy, although higher ploidy levels are very rare for insects except for some weevils [3]. In the instance of terminal fusion in *H. haemorrhoidalis*, hexaploidy may be the more likely ploidy level than triploidy, with different expectations for allelic diversity within individuals (i.e. three pairs of ‘diploid loci’ which have become ‘homozygous’ through terminal fusion). Recently, Jacobson *et al.* [75] have detected variation in ploidy between arrhenotokous and thelytokous lineages of *T. tabaci* by using flow cytometry combined with the microsatellite analyses. Among five thelytokous isofemale lines of *T. tabaci*, one line was considered diploid while four lines were polyploid, also with a maximum of three alleles per locus observed in seven microsatellite loci. Given that all analysed *H. haemorrhoidalis* populations were thelytokous we would not expect variation in ploidy levels of this species. Combined, our study and Jacobson *et al*. [75] provide exciting new insights and warrant future research into the mechanism and evolution of thelytoky in Thysanoptera and its potential link to polyploidy [3].

### *Low genetic diversity and the origin of thelytoky in* H. haemorrhoidalis

We did not find any variation in *COI* of 27 thrips individuals and only low diversity in *H3* and *EF1a* of nine individuals collected from five continents*.* It is possible that the species experienced genetic bottlenecks prior to (e.g. through a switch to thelytokous reproduction) or early in the invasion process. Unfortunately, the overall low genetic diversity and our small sample number does not allow us to test the hypothesis of a South American origin of this species, also as we were not able to obtain samples from its potential region of origin in the Amazon region [53]. Future studies should look more intensively into this region, also as the thrips diversity of some South American regions remain poorly explored [22].

It has previously been suggested that parthenogenetic species normally originate from their sexual ancestors [77]. Male *H. haemorrhoidalis* recorded from South America may indicate that sexual reproduction of *H. haemorrhoidalis* perhaps still occurs in its native range, together with thelytokous populations [26]. Thelytokous populations may have experienced a reproductive advantage over sexual populations and then spread throughout the world. Alternatively, this species may have lost its capacity to reproduce sexually and only exists in its parthenogenetic form with occasional production of males in some regions [78]. Previous analyses that revealed absence of spermatheca in females of *H. haemorrhoidalis* would support such a loss of sexual functionality in this species [79], and rare males observed in thelytokous populations may result from a failure in diploidy restoration [80]. The elucidation of *H. haemorrhoidalis*’s origin and its thelytokous evolution will require further studies of genetic diversity, particularly in the likely region of origin of this species, as well as cytological studies.

## Conclusions

In our study we were unable to confirm earlier reports of *Wolbachia* infections in *H. haemorrhoidalis*, yet this previous report was perhaps prematurely discussed as mechanism of thelytoky in this species [11,12,32-34]. We have confirmed that thelytoky in this species is due to genetic factors in the host genome, and in the absence of endosymbiotic bacteria, as antibiotic treatment did not result in the reproduction of males. The analysis of genetic diversity at two nuclear loci has indicated that *H. haemorrhoidalis* may be polyploid (and potentially hexaploid) which does warrant future cytogenetic investigation, in particular as this species is known to reproduce by automixis through terminal fusion. This species may be an important model for unravelling the cytological mechanisms behind thelytokous parthenogenesis in Thysanoptera, and in insects in general.

## Availability of supporting data

The data sets supporting the results of this article are included within the article and its additional files. All DNA sequences were deposited and GenBank accession numbers were listed in Additional file 6: Table S6 and Additional file 7: Table S7.

## Additional files

Additional file 1: Table S1. Collection details of *H. haemorrhoidalis* and other thrips specimens. Additional file 2: Table S2. Primer sequences used in this study. Additional file 3: Table S3. Primer sequences used for nested PCR to detect *Wolbachia*. Additional file 4: Table S4. PCR master mix concentrations. Additional file 5: Table S5. PCR thermocycling conditions. Additional file 6: Table S6. Clone sequences of 16S rDNA isolated from *H. haemorrhoidalis.* Two different types of primer pairs were used. One to four isolates per ligated and cloned PCR product were sequenced. Some BLAST matches were also with chloroplast 16S rDNA from *Citrus sinensis* (in bold). Additional file 7: Table S7. GenBank accession numbers of *COI*, *H3* and *EF1a* sequences of *H. haemorrhoidalis* and other thrips species. Additional file 8: Table S8. Number of DNA extracts (n) of laboratory and field individuals tested for *Cardinium* (C) and *Wolbachia* (W). Extracts were single or pooled (1, 5, 10 or 20) individuals. Australian individuals were from Richmond (New South Wales NSW), Canberra (Australian Capital Territory ACT) and Sunshine Beach (Queensland QLD). Additional file 9: Figure S1. Mean number of female progeny of *H. haemorrhoidalis* (n= 10) after treatment of adult specimens (experiment 1) with different concentrations of rifampicin and tetracycline hydrochloride. Treatments did not result in reproduction of males. For 2.5% and 5% tetracycline hydrochloride, all treated mothers died after the 2^nd^ week, and there was no offspring in the 3^rd^ week after treatment. Additional file 10: Figure S2. Phylogenetic tree based on the *COI* gene (627bp) of *H. haemorrhoidalis* and other thrips species within the family of Thripidae constructed by Bayesian Inference (model GTR+G+I). *Haplothrips victoriensis* (suborder Tubulifera: Phlaeothripidae) was used as outgroup. Numbers at nodes represent posterior probabilities >50%. (*) One identical sequence was amplified from all 27 individuals from this study. Scale bar represents the number of nucleotide substitutions per site. Additional file 11: Figure S3. Polymorphic sites within exons and introns in an *H3* gene fragment (453bp) defined the different alleles of *H. haemorrhoidalis.* Individuals had at least two different alleles (maximum of three). The intron started with GTA (position 210-212) and ended with CAG (position 330-332). The exon of all alleles was 330bp in length. Indels are represented by a dash, while a match is represented by a point. Additional file 12: Figure S4. Polymorphic sites within exons and introns in a *EF1a* gene fragment (861bp) defined different alleles of *H. haemorrhoidalis* (allele names in the last column). Individuals had at least two alleles or a maximum of three alleles. The first intron started with GTA (position 154-56) and ended with TAG (position 224-226). The second intron started with GTA (624-626) and ended with TAG (728-730); intron 2 was variable in size; alleles A2, A3 and A9 were 83bp, while A1, A4, A5, A6, A7, A8, A11 and A12 were 85bp long; allele A10 was 101bp long. The exon sequence was 681bp long. Indels are represented by a dash, while a match is represented by a point. Additional file 13: Figure S5.
*Heliothrips haemorrhoidalis* phylogenetic tree of *EF1a* allelic sequences (including intron) constructed with Bayesian likelihood inference (Model T92+G). Allele numbers A1-A12 are represented by collection locations. Numbers at nodes represent posterior probabilities >50%. Scale bar represents the number of nucleotide substitutions per site.
