# Supplementary material for: Polyploidy versus endosymbionts in obligately thelytokous thrips
Source: BMC Evol Biol. 2015 Feb 22;15:23. doi: 10.1186/s12862-015-0304-6 (PMC4349774; doi:10.1186/s12862-015-0304-6)
Supplement: Additional file 1: Table S1. — Collection details of H. haemorrhoidalis and other thrips specimens. [file 12862_2015_304_MOESM1_ESM.doc]

**Additional file 1: Table S1. Collection details of *Heliothrips haemorrhoidalis* and other thrips specimens.**

| **#** | **Location** | **Year** | **Specimen source** | **Number of females** | **Number of males** | **Collector** | **Host plants** |
| --- | --- | --- | --- | --- | --- | --- | --- |
| 1. ***Heliothrips haemorrhoidalis*** | | | | | | | |
| 1 | Richmond, NSW, Australia | 1995 | Laboratory population [35] | 1,500 | 0 | Robert  Spooner-Hart ^(1)^ | Citrus |
| 2 | Canberra, ACT, Australia | 2011 | Laboratory population | 2,000 | 0 | Robert  Spooner-Hart | *Viburnum* sp. |
| 3 | Sunshine Beach, Queensland, Australia | 2009 | Field | 3 | 0 | Desley J. Tree^(2)^ | *Persoonia stradbrokensis* |
| 4 | Te Puke, New Zealand | 2013 | Laboratory population | 50 | 0 | Christina Rowe^(3)^ | Myer lemons |
| 5 | Western Cape, South Africa | 2012 | Field | 20 | 0 | Michael Stiller ^(4)^ | Persimmon |
| 6 | Higashihiroshima, Hiroshima, Japan | 1995, 1997 | Field | 10 | 0 | Satoshi Toda ^(5)^ | Kiwifruit  Bitter orange |
| 7 | Valencia, Spain | 2012 | Field | 50 | 0 | Cristina Navarro Campos^(6)^ | *Viburnum tinus* |
| 8 | Kew Gardens, United Kingdom | 2014 | Field | 10 | 0 | Alison  Scott-Brown^(7)^ | Glasshouse |
| 9 | Nogales, Valparaiso Region, Chile | 2012 | Field | 120 | 0 | Renato Ripa ^(8)^ | Avocado |
| 1. **Other thrips species** | | | | | | | |
| 1 | New Zealand | 2011 | Laboratory population | *Frankliniella occidentalis* | | Grant Herron^(9)^ | Cucumbers |
| 2 | UWS Hawkesbury, Richmond, NSW, Australia | 2013 | Field | *Pezothrips kellyanus* | | Duong Nguyen^(1)^ | Myer lemons |
| 3 | South Australia, Australia | 2012 | Field | *Thrips imaginis* | | Greg Baker^(10)^ | Roses |
| 4 | Collembally, NSW, Australia | 2009 | Field | *Thrips tabaci* | | Jianghua Mo^(11)^ | Onions |

*Notes:*

*^(1)^ University of Western Sydney, Australia*

*^(2)^ Queensland Department of Agriculture, Fisheries and Forestry, Australia*

*^(3)^ The New Zealand Institute for Plant & Food Research Limited, New Zealand*

*^(4)^ Biosystematics Division, Agricultural Research Council, Plant Protection Research Institute, South Africa*

*^(5)^ National Institute of Fruit Tree Science, NARO, Japan.*

*^(6)^ Institut Agroforestal Mediterrani, Universitat Politècnica de València, Spain*

*^(7)^ The Royal Botanic Gardens, Kew, United Kingdom*

*^(8)^ BioCEA-Chile*

*^(9)^ NSW Department of Primary Industries, Australia*

*^(10)^* *South Australian Research and Development Institute, Australia*

*^(11)^* *Yanco Agricultural Institute Industries & Investments NSW, Australia*
