# Supplementary material for: Polyploidy versus endosymbionts in obligately thelytokous thrips
Source: BMC Evol Biol. 2015 Feb 22;15:23. doi: 10.1186/s12862-015-0304-6 (PMC4349774; doi:10.1186/s12862-015-0304-6)
Supplement: Additional file 2: Table S2. — Primer sequences used in this study. [file 12862_2015_304_MOESM2_ESM.doc]

**Additional file 2:** **Table S2.** Primer sequences used in this study.

| **Primer name** | **Primer sequence (5’-3’)** | **Target gene** | **Reference** |
| --- | --- | --- | --- |
| 16SWfor | TTGTAGCCTGCTATGGTATAACT | 16SWrRNA | [41] |
| 16SWrev | GAATAGGTATGATTTTCATGT |  |  |
| WspecF | CATACCTATTCGAAGGGATAG | 16SWrRNA | [40] |
| WspecR | AGCTTCGAGTGAAACCAATTC |  |  |
| 553F_W | CTTCATRYACTCGAGTTGCWGAGT | 16SWrRNA | [45] |
| 1334R_W | GAKTTAAAYCGYGCAGGBGTT |  |  |
| ftsZF1 | GTTGTCGCAAATACCGATGC | *ftsZ* | [44] |
| ftsZR1 | CTTAAGTAAGCTGGTATATC |  |  |
| ftsZ102_FOR | AATGCTGTGAATAACATGATGAT | *ftsZ* | [43] |
| ftsZ969_REV | CCAGTTGCAAGAACAGAAAC |  |  |
| 81F | TGGTCCAATAAGTGATGAAGAAAC | *wsp* | [39] |
| 691R | AAAAATTAAACGCTACTCCA |  |  |
| Wsp for | TGGTCCAATAAGTGATGAAGAAACTAGCTA | *wsp* | [47] |
| Wsp rev | AAAAATTAAACGCTACTCCAGCTTCTGCAC |  |  |
| gatB_F1 | GAKTTAAAYCGYGCAGGBGTT | *gatB*-MLST | [42] |
| gatB_R1 | TGGYAAYTCRGGYAAAGATGA |  |  |
| coxA_F1 | TTGGRGCRATYAACTTTATAG | *coxA*-MLST |  |
| coxA_R1 | CTAAAGACTTTKACRCCAGT |  |  |
| hcpA_F1 | GAAATARCAGTTGCTGCAAA | *hcpA*-MLST |  |
| hcpA_R1 | GAAAGTYRAGCAAGYTCTG |  |  |
| ftsZ_F1 | ATYATGGARCATATAAARGATAG | *ftsZ*-MLST |  |
| ftsZ_R1 | TCRAGYAATGGATTRGATAT |  |  |
| fbpA_F1 | GCTGCTCCRCTTGGYWTGAT | *fpbA*-MLST |  |
| fbpA_R1 | CCRCCAGARAAAAYYACTATTC |  |  |
| wsp_R1 | CYGCACCAAYAGYRCTRTAAA | *wsp*-MLST |  |
| wsp_F1 | GTCCAATARSTGATGARGAAAC |  |  |
| ChF | TACTGTAAGAATAAGCACCGGC | *Cardinium* | [15] |
| ChR | GTGGATCACTTAACGCTTTCG |  |  |
| CLOf1 | GGAACCTTACCTGGGCTAGAATGTATT | *Cardinium* | [48] |
| CLOr1 | GCCACTGTCTTCAAGCTCTACCAAC |  |  |
| LCO1490 | GGTCAACAAATCATAAAGATATTGG | *COI* | [52] |
| HCO2198 | TAAACTTCAGGGTGACCAAAAAATCA |  |  |
| H3AF | ATGGCTCGTACCAAGCAGACVGC | *Histone 3 H3* | [54] |
| H3AR | ATATCCTTRGGCATRATRGTGAC |  |  |
| EF1aF-Hh | ATCTCTGGTTGGCATGGAGACAA | *Elongation factor 1α* | This study |
| rcM4-Hh | ACAGCCACGGTCTGTCTCATGTC |  | This study |

Notes: MLST: Multi Locus Sequence Typing System for *Wolbachia* developed by Baldo *et al*. [42]
