# Supplementary material for: Polyploidy versus endosymbionts in obligately thelytokous thrips
Source: BMC Evol Biol. 2015 Feb 22;15:23. doi: 10.1186/s12862-015-0304-6 (PMC4349774; doi:10.1186/s12862-015-0304-6)
Supplement: Additional file 3: Table S3. — Primer sequences used for nested PCR to detect Wolbachia. [file 12862_2015_304_MOESM3_ESM.doc]

**Additional file 3:** **Table S3.** Primer sequences used for nested PCR to detect *Wolbachia*.

| **PCR** | **Primer name** | **Primer sequence (5’-3’)** | **Target gene** | **Reference** |
| --- | --- | --- | --- | --- |
| 1^st^run | 61F | GCTTAACACATGCAAG | 16S rRNA | [41] |
|  | 1227R | CCATTGTAGCACGTGT |  |  |
|  | 10F | AGTTTGATCATGGCTCAGATTG |  |  |
|  | 1507R | TACCTTGTTACGACTTCACCCCAG |  |  |
| 2^nd^run | 16SWfor | TTGTAGCCTGCTATGGTATAACT |  |  |
|  | 16SWrev | GAATAGGTATGATTTTCATGT |  |  |
| 1^st^run | 81F | TGGTCCAATAAGTGATGAAGAAAC | *Wsp* | [39] |
|  | 691R | AAAAATTAAACGCTACTCCA |  |  |
| 2^nd^run | 136F | TGAAATTTTACCTCTTTTC | *Wsp*  supergroup A specific |  |
|  | 691R | AAAAATTAAACGCTACTCCA |  |  |
| 1^st^run | 81F | TGGTCCAATAAGTGATGAAGAAAC | *Wsp* |  |
|  | 691R | AAAAATTAAACGCTACTCCA |  |  |
| 2^nd^run | 81F | TGGTCCAATAAGTGATGAAGAAAC | *Wsp*  supergroup B specific |  |
|  | 522R | ACCAGCTTTTGCTTGATA |  |  |
| 1^st^run | gatB_F3 | ATTCAYYTAGARCAAGATGCAGG | *gatB*-MLST | (*) |
|  | gatB_R3 | AAGAGCTCKGAYAAAGCATYBGC |  |  |
| 2^nd^run | gatB_F1 | GAKTTAAAYCGYGCAGGBGTT |  |  |
|  | gatB_R1 | TGGYAAYTCRGGYAAAGATGA |  |  |
| 1^st^run | coxA_F3 | ATGATTGGCKCACCHGAYATGGC | *coxA*-MLST |  |
|  | coxA_R3 | ACTTTTACACCAGTWATMACRCC |  |  |
| 2^nd^run | coxA_F1 | TTGGRGCRATYAACTTTATAG |  |  |
|  | coxA_R1 | CTAAAGACTTTKACRCCAGT |  |  |
| 1^st^run | hcpA_F3 | ATTAGAGAAATARCAGTTGCTGC | *hcpA*-MLST |  |
|  | hcpA_R3 | CATGAAAGACGAGCAARYTCTGG |  |  |
| 2^nd^run | hcpA_F1 | GAAATARCAGTTGCTGCAAA |  |  |
|  | hcpA_R1 | GAAAGTYRAGCAAGYTCTG |  |  |
| 1^st^run | fbpA_F3 | GTTAACCCTGATGCYYAYGAYCC | *fpbA*-MLST |  |
|  | fbpA_R3 | TCTACTTCCTTYGAYTCDCCRCC |  |  |
| 2^nd^run | fbpA_F1 | GCTGCTCCRCTTGGYWTGAT |  |  |
|  | fbpA_R1 | CCRCCAGARAAAAYYACTATTC |  |  |
| 1^st^run | ftsZunif | GG(CT)AA(AG)GGTGC(AG)GCAGAAGA | *ftsZ*-MLST |  |
|  | ftsZunir | ATC(AG)AT(AG)CCAGTTGCAAG |  |  |
| 2^nd^run | ftsZ_F1 | ATYATGGARCATATAAARGATAG |  |  |
|  | ftsZ_R1 | TCRAGYAATGGATTRGATAT |  |  |

(*)<http://pubmlst.org/wolbachia/info/amp_seq_single.shtml>

Note: Nested PCR for 16Sr DNA and MLST loci can be used to detect all *Wolbachia* supergroups.
