# Supplementary material for: Polyploidy versus endosymbionts in obligately thelytokous thrips
Source: BMC Evol Biol. 2015 Feb 22;15:23. doi: 10.1186/s12862-015-0304-6 (PMC4349774; doi:10.1186/s12862-015-0304-6)
Supplement: Additional file 4: Table S4. — PCR master mix concentrations. [file 12862_2015_304_MOESM4_ESM.doc]

**Additional file 4:** **Table S4.** PCR master mix concentrations.

| **Primers** | **MgCl_2_ (mM)** | **10X buffer** | **Fwd primer (µM)** | **Rev primer (µM)** | **dNTPs (mM)** | **Taq**^(1)^ **(U)** | **Template (µL)** | **FV (µL)** |
| --- | --- | --- | --- | --- | --- | --- | --- | --- |
| 16SWfor - 16SWrev | 2 | 1 | 0.6 | 0.6 | 0.2 | 1 | 1µL; 2µL ^(2)^ | 25 |
| WspecF - WspecR | 2 | 1 | 0.63 | 0.63 | 0.125 | 0.5 | 1µL; 2µL^(2)^ | 20 |
| 553F_W- 1334R_W | 1.5 | 1 | 0.2 | 0.2 | 2 | 0.25 | 1µL; 2µL^(2)^ | 20 |
| ftsZF1 - ftsZR1 | 1.25 | 1 | 0.5 | 0.5 | 0.02 | 0.5 | 1µL; 2µL^(2)^ | 20 |
| ftsZ102 FOR - ftsZ969 REV | 1.5 | 1 | 0.75 | 0.75 | 0.2 | 1.5 | 1µL; 2µL^(2)^ | 20 |
| ftsZ102 FOR – ftsZ969 REV ^(3)^ | 1.5 | 1 | 0.75 | 0.75 | 0.8 | 1.5 | 1µL; 2µL^(2)^ | 20 |
| 81F - 691R | 2.5 | 1 | 0.5 | 0.5 | 0.25 | 1 | 2µL | 20 |
| 136F - 691R | 2.5 | 1 | 0.5 | 0.5 | 0.25 | 1 | 2µL | 20 |
| 81F - 522R | 2.5 | 1 | 0.5 | 0.5 | 0.25 | 1 | 2µL | 20 |
| Wsp for - Wsp rev | 4 | 1 | 0.8 | 0.8 | 0.15 | 0.8 | 2µL | 20 |
| MLST F1-R1 | 1.5 | 1 | 1 | 1 | 0.2 | 0.5 | 2µL | 20 |
| MLST F3-R3 | 1.5 | 1 | 0.5 | 0.5 | 0.2 | 0.5 | 2µL | 20 |
| ftsZunif - ftsZunir | 2.5 | 1 | 0.5 | 0.5 | 0.25 | 1 | 2µL | 20 |
| ChF - ChR | 3 | 1 | 0.8 | 0.8 | 1 | 1.5 | 2µL | 20 |
| CLOf1 - CLOr1 | 3 | 1 | 0.8 | 0.8 | 1 | 1.5 | 2µL | 20 |
| 61F - 1227R | 2.5 | 1 | 0.4 | 0.4 | 0.2 | 1 | 2µL | 20 |
| 10F - 1507R | 2.5 | 1 | 0.4 | 0.4 | 0.2 | 1 | 2µL | 20 |
| LCO1490 - HCO2198 ^(4)^ | 2 | 1 | 0.2 | 0.2 | 0.02 | 1 | 2µL | 25 |
| H3AF - H3AR | 3 | 1 | 0.8 | 0.8 | 1 | 1.5 | 2µL | 20 |
| EF1aF-Hh - rcM4-Hh | 2.5 | 1 | 0.4 | 0.4 | 0.2 | 1 | 2µL | 20 |
| SP6 - T7 promoter | 2.5 | 1 | 0.4 | 0.4 | 0.2 | 1 | 2µL | 20 |

## *Abbreviations:* Fw primer: Forward primer; Rv primer: Reverse primer; FV: Final volume

## (1) GoTaq DNA polymerase (Promega) was used for all experiments

## (2) Two PCR assays were performed to test two different volumes of DNA: 1µL; 2µL

## (3) PCR protocol used in Pintureau *et al.* [31]

## (4): 125ng BSA: Bovine Serum Albumin (New England Biolabs) was added
