# Supplementary material for: Polyploidy versus endosymbionts in obligately thelytokous thrips
Source: BMC Evol Biol. 2015 Feb 22;15:23. doi: 10.1186/s12862-015-0304-6 (PMC4349774; doi:10.1186/s12862-015-0304-6)
Supplement: Additional file 5: Table S5. — PCR thermocycling conditions. [file 12862_2015_304_MOESM5_ESM.doc]

**Additional file 5:** **Table S5.** PCR thermocycling conditions.

| **Loci** | **Step 1** | | **Step 2** | | **Step 3** | | **Step 4** | | **Number of cycles** | **Step 5** | | **Step 6** | | **Step 7** | | **Number of cycles** | **Step 8** | |
| --- | --- | --- | --- | --- | --- | --- | --- | --- | --- | --- | --- | --- | --- | --- | --- | --- | --- | --- |
|  | T | D | T | D | T | D | T | D |  | T | D | T | D | T | D |  | T | D |
| 16SWfor - 16SWrev | 94 | 180 | 94 | 30 | 52 | 30 | 72 | 90 | 35 | 72 | 600 |  |  |  |  |  |  |  |
| WspecF - WspecR | 95 | 120 | 95 | 30 | 54 | 30 | 72 | 60 | 35 | 72 | 600 |  |  |  |  |  |  |  |
| 553F_W- 1334R_W | 94 | 120 | 94 | 60 | 62 | 60 | 72 | 60 | 35 | 72 | 600 |  |  |  |  |  |  |  |
| ftsZF1 - ftsZR1(Werren et al., 1995) | 94 | 180 | 94 | 30 | 55 | 30 | 72 | 60 | 35 | 72 | 600 |  |  |  |  |  |  |  |
| ftsZ102_FOR - ftsZ969_REV  (Holden et al., 1993) | 94 | 180 | 94 | 30 | 50 | 30 | 72 | 90 | 35 | 72 | 600 |  |  |  |  |  |  |  |
| ftsZ102_FOR - ftsZ969_REV (*)  (Holden et al., 1993) | 94 | 180 | 94 | 30 | 51 | 45 | 72 | 45 | 35 | 72 | 600 |  |  |  |  |  |  |  |
| 81F - 691R | 94 | 180 | 94 | 30 | 55 | 30 | 72 | 60 | 35 | 72 | 600 |  |  |  |  |  |  |  |
| 136F - 691R | 94 | 180 | 94 | 30 | 55 | 30 | 72 | 60 | 35 | 72 | 600 |  |  |  |  |  |  |  |
| 81F - 522R | 94 | 180 | 94 | 30 | 55 | 30 | 72 | 60 | 35 | 72 | 600 |  |  |  |  |  |  |  |
| Wsp for - Wsp rev | 94 | 120 | 94 | 10 | 65 | 30 | 68 | 60 | 9 | 94 | 10 | 65 | 30 | 68 | 60 | Cycle step 5 for 24 times and increase 20sec every cycle |  |  |
| MLST F1-R1 | 94 | 120 | 94 | 30 | Primer specific T | 45 | 72 | 90 | 37 | 72 | 600 |  |  |  |  |  |  |  |
| MLST F3-R3 | 95 | 300 | 95 | 15 | Primer specific T | 30 | 72 | 90 | 35 | 72 | 600 |  |  |  |  |  |  |  |
| ftsZunif - ftsZunir | 94 | 180 | 94 | 30 | 55 | 30 | 72 | 80 | 39 | 72 | 600 |  |  |  |  |  |  |  |
| ChF - ChR | 94 | 180 | 94 | 30 | 55 | 30 | 72 | 60 | 35 | 72 | 600 |  |  |  |  |  |  |  |
| CLOf1 - CLOr1 | 94 | 180 | 94 | 30 | 54 | 30 | 72 | 60 | 35 | 72 | 600 |  |  |  |  |  |  |  |
| 61F - 1227R | 94 | 180 | 94 | 30 | 53 | 30 | 72 | 105 | 35 | 72 | 600 |  |  |  |  |  |  |  |
| 10F - 1507R | 94 | 180 | 94 | 30 | 53 | 30 | 72 | 105 | 35 | 72 | 600 |  |  |  |  |  |  |  |
| LCO1490 - HCO2198 | 94 | 60 | 94 | 30 | 45 | 90 | 72 | 60 | 5 | 94 | 30 | 51 | 90 | 72 | 60 | Cycle step 5 for 35times | 72 | 300 |
| H3AF - H3AR | 94 | 120 | 94 | 30 | 52 | 30 | 72 | 45 | 35 | 72 | 420 |  |  |  |  |  |  |  |
| EF1aF-Hh - rcM4-Hh | 95 | 180 | 95 | 30 | 56 | 30 | 72 | 60 | 39 | 72 | 600 |  |  |  |  |  |  |  |
| SP6 - T7 promoter | 94 | 180 | 94 | 30 | 50 | 30 | 72 | 90 | 35 | 72 | 600 |  |  |  |  |  |  |  |

**Abbreviations:**

T: temperature (**°**C)

D: Duration (seconds)

MLST F1-R1: primers of Multi Locus Sequence Typing System

MLST F3-R3: nested PCR primers of Multi Locus Sequence Typing System

Primer specific T: annealing temperature according to specific primers:

| - *hcpA*F1-R1: 53**°**C; - *coxA* F1-R1: 55**°**C; - *fpbA* F1-R1: 59**°**C; - *gatB* F1-R1: 54**°**C; - *ftsZ* F1-R1: 50**°**C | - *hcpA* F3-R3: 50**°**C; - *coxA* F3-R3: 52**°**C; - *fpbA* F3-R3: 50**°**C; - *gatB* F3-R3: 50**°**C; |
| --- | --- |

## (*) PCR protocol used in Pintureau *et al.* [31]
