# Supplementary material for: Polyploidy versus endosymbionts in obligately thelytokous thrips
Source: BMC Evol Biol. 2015 Feb 22;15:23. doi: 10.1186/s12862-015-0304-6 (PMC4349774; doi:10.1186/s12862-015-0304-6)
Supplement: Additional file 6: Table S6. — Clone sequences of 16S rDNA isolated from H. haemorrhoidalis. Two different types of primer pairs were used. One to four isolates per ligated and cloned PCR product were sequenced. Some BLAST matches were also with chloroplast 16S rDNA from Citrus sinensis (in bold). [file 12862_2015_304_MOESM6_ESM.doc]

**Additional file 6: Table S6. Clone sequences of 16S rDNA isolated from *Heliothrips haemorrhoidalis.* Two different types of primer pairs were used. One to four isolates per ligated and cloned PCR product were sequenced. Some BLAST matches were also with chloroplast 16S rDNA from *Citrus sinensis* (in bold).**

| **Year** | **Primers** | **Extracts (Plate)** | **Cloned sequences** | **GenBank** | **BLAST analysis results:** bacteria and **chloroplasts (in bold)** |
| --- | --- | --- | --- | --- | --- |
| 2011 | 10F-1507R | Richmond - 1 larva (Plate 2) | 2-1 | KM582833 | Uncultured bacterium: HM556384.1  *Zymobacter palmae*: NR_041786.1  Bacterial symbiont of *Bactrocera correcta:* JQ950499.1 |
|  |  |  | 2-3 | KM582834 |  |
|  | 61F-1227R | Canberra -1 adult (Plate 3) | 3-1 | KM582835 | *Acinetobacter* sp.: KC257041.1 Uncultured soil bacterium:  HM131962.1 *Acinetobacter* sp. FJ815433.1 |
|  |  |  | 3-2 | KM582836 | *Pectobacterium cypripedii:* [HM013841.1](http://www.ncbi.nlm.nih.gov/nucleotide/302180058?report=genbank&log$=nucltop&blast_rank=1&RID=YJZDZZDW01R) Uncultured bacterium: HM559142.1*Erwinia* sp.: HM748063.1Uncultured *Erwinia* sp. from *Frankliniella schultzei*: JN793861.1 |
|  |  | Richmond - 1 larva (Plate 4) | 5-1 | KM582837 | ***Citrus sinensis* chloroplast: DQ864733.1**  Uncultured bacterium clone: KF068101.1 |
|  |  | Richmond - 1 adult - weak band (Plate 5) | 5-1-1 | KM582838 | Uncultured bacteria from a leaf-cutter ant nest: HM558614.1; HM557934.1 |
|  |  | Richmond - 1 larva - weak band (plate 6) | 6-3 | KM582839 |  |
| 2013 | 61F-1227R | Richmond- 1 adult – (plate 1) | Hh1-1 | KM582840 | ***Citrus sinensis* chloroplast:** [**DQ864733.1**](http://www.ncbi.nlm.nih.gov/nucleotide/113952601?report=genbank&log$=nucltop&blast_rank=12&RID=YK30XW2R014)  Uncultured bacterium: KF084751.1 |
|  |  |  | Hh1-2 | KM582841 | Uncultured bacteria:  JQ410853.1; JX680760.1; KF079943.1  *Serratia* sp. endosymbiont of *Nilaparvata lugens:* GU124496.1 |
|  |  |  | Hh1-3 | KM582842 | ***Citrus sinensis* chloroplast:** [**DQ864733.1**](http://www.ncbi.nlm.nih.gov/nucleotide/113952601?report=genbank&log$=nucltop&blast_rank=12&RID=YK30XW2R014)  Uncultured bacterium: KF084751.1 |
|  |  |  | Hh1-4 | KM582843 |  |
|  |  | Richmond - 1 adult- (plate 2) | Hh2-1 | KM582844 | Enterobacteriaceae bacterium: [EU029106.1](http://www.ncbi.nlm.nih.gov/nucleotide/152938356?report=genbank&log$=nucltop&blast_rank=3&RID=YK6SSGXP014)  Unidentified gut bacterium thrips: AF024609.1 |
|  |  |  | Hh2-2 | KM582845 | Enterobacteriaceae bacterium stinkbugs and other arthropods:  JQ726781.1  Unidentified gut bacterium thrips: AF024609.1  *Enterobacter agglomerans*: AF024608.1_ |
|  |  |  | Hh2-3 | KM582846 | Gluconacetobacter: NR_114384.1; AY961985.1  *Asaia astilbis:* AB485744.1  *Gluconacetobacter europaeus*: EU096233.1  *Gluconobacter oxydans*: AF127396.1 Uncultured bacterium: HM557085.1 |
|  |  |  | Hh2-4 | KM582847 | *Frankliniella bispinosa*: JN793838.1  *Frankliniella occidentalis*_BFo-1: EU029105.1  Enterobacteriaceae bacterium: [EU029106.1](http://www.ncbi.nlm.nih.gov/nucleotide/152938356?report=genbank&log$=nucltop&blast_rank=3&RID=YK6SSGXP014) |
|  |  | Chile - 1 adult – (plate 3) | Hh3-1 | KM582848 | Uncultured bacteria: [KF095246.1](http://www.ncbi.nlm.nih.gov/nucleotide/643415552?report=genbank&log$=nucltop&blast_rank=1&RID=YNKYA6NJ01R), KF843326.1Uncultured eukaryote clone: GQ080992.1 |
|  |  |  | Hh3-2 | KM582849 |  |
|  |  |  | Hh3-3 | KM582850 |  |
|  |  |  | Hh3-4 | KM582851 |  |
| **Total of sequenced clones** | | | **19** |  |  |

*Notes: The following sequences were identical or very similar:*

5-1-1 and 6-3 5-1-1 and 6-3; Hh3-1 Hh3-2, Hh3-3 and Hh3-4 Hh2-1, Hh2-2 and Hh2-4
