# Supplementary material for: Polyploidy versus endosymbionts in obligately thelytokous thrips
Source: BMC Evol Biol. 2015 Feb 22;15:23. doi: 10.1186/s12862-015-0304-6 (PMC4349774; doi:10.1186/s12862-015-0304-6)
Supplement: Additional file 7: Table S7. — GenBank accession numbers of COI, H3 and EF1a sequences of H. haemorrhoidalis and other thrips species. [file 12862_2015_304_MOESM7_ESM.doc]

**Additional file 7:** **Table S7.** GenBank accession numbers of *Heliothrips haemorrhoidalis* and other thrips species.

| **Location** | ***COI*** | ***H3*** | | ***EF1a*** | |
| --- | --- | --- | --- | --- | --- |
|  | **GenBank** | **Alleles** | **GenBank** | **Alleles** | **GenBank** |
| **a) *Heliothrips haemorrhoidalis*** | | |  |  |  |
| Australia,  Richmond | KM582797 | n.d. | n.d. | n.d. | n.d. |
| Australia, ACT | KM582798 | A1 | KM582852 | A1 | KM582810 |
|  |  | A2 | KM582853 | A2 | KM582821 |
|  |  |  |  |  |  |
| Australia, Queensland | KM582799 | A1 | KM582855 | A2 | KM582822 |
|  |  | A3 | KM582854 | A3 | KM582823 |
|  |  | A5 | KM582856 | A4 | KM582811 |
|  |  |  |  |  |  |
| New Zealand | KM582800 | A1 | KM582857 | A2 | KM582824 |
|  |  | A2 | KM582858 | A5 | KM582812 |
|  |  |  |  |  |  |
| South Africa | KM582801 | A1 | KM582860 | A2 | KM582825 |
|  |  | A3 | KM582859 | A6 | KM582813 |
|  |  | A4 | KM582861 | A7 | KM582814 |
|  |  |  |  |  |  |
| Japan | KM582802 | A1 | KM582862 | A2 | KM582826 |
|  |  | A4 | KM582863 | A5 | KM582815 |
|  |  | - | - | A8 | KM582816 |
|  |  |  |  |  |  |
| United Kingdom | KM582804 | A1 | KM582864 | A2 | KM582828 |
|  |  | A4 | KM582865 | A5 | KM582817 |
|  |  | - | - | A9 | KM582827 |
|  |  |  |  |  |  |
| Spain | KM582803 | A1 | KM582868 | A2 | KM582829 |
|  |  | A2 | KM582867 | - | - |
|  |  | A4 | KM582866 | - | - |
|  |  |  |  |  |  |
| Chile 1 | KM582805 | A1 | KM582870 | A2 | KM582831 |
|  |  | A3 | KM582869 | A6 | KM582818 |
|  |  | A4 | KM582871 | A10 | KM582830 |
|  |  |  |  |  |  |
| Chile 2 | KM582805 | n.d. | n.d. | A2 | KM582832 |
|  |  | n.d. | n.d. | A11 | KM582819 |
|  |  | n.d. | n.d. | A12 | KM582820 |
| **b) Other thrips species** | |  |  |  |  |
| Australia | n.d. | n.d. | n.d. | *Pezothrips kellyanus* | KM582806 |
| New Zealand | n.d. | n.d. | n.d. | *Frankliniella occidentalis* | KM582807 |
| Australia | n.d. | n.d. | n.d. | *Thrips imaginis* | KM582808 |
| Australia | n.d. | n.d. | n.d. | *Thrips tabaci* | KM582809 |

n.d.: not determined
