# Supplementary material for: Polyploidy versus endosymbionts in obligately thelytokous thrips
Source: BMC Evol Biol. 2015 Feb 22;15:23. doi: 10.1186/s12862-015-0304-6 (PMC4349774; doi:10.1186/s12862-015-0304-6)
Supplement: Additional file 8: Table S8. — Number of DNA extracts (n) of laboratory and field individuals tested for Cardinium (C) and Wolbachia (W). Extracts were single or pooled (1, 5, 10 or 20) individuals. Australian individuals were from Richmond (New South Wales NSW), Canberra (Australian Capital Territory ACT) and Sunshine Beach (Queensland QLD). [file 12862_2015_304_MOESM8_ESM.doc]

**Additional file 8:** **Table S8.** Number of DNA extracts (n) of laboratory and field individuals tested for *Cardinium* (C) and *Wolbachia* (W). Extracts were single or pooled (1, 5, 10 or 20) individuals. Australian individuals were from Richmond (New South Wales NSW), Canberra (Australian Capital Territory ACT) and Sunshine Beach (Queensland QLD).

| **#** | **Location** | | **Number of adults per DNA extract** | | | | | | **Number of pupae per DNA extract** | | | **Number of larvae per DNA extract** | | | | **Total** | |
| --- | --- | --- | --- | --- | --- | --- | --- | --- | --- | --- | --- | --- | --- | --- | --- | --- | --- |
|  |  |  | **1** | | **5** | **10** | | **20** | **1** | **10** | **20** | **1** | | **10** | **20** | ***C*** | ***W*** |
|  |  |  | ***C*** | ***W*** | ***W*** | ***C*** | ***W*** | ***W*** | ***W*** | ***W*** | ***W*** | ***C*** | ***W*** | ***W*** | ***W*** |  |  |
| **Laboratory populations** | | n extracts | **27** | **70** | **6** | **2** | **10** | **6** | **2** | **2** | **2** | **3** | **11** | **8** | **6** | **32** | **123** |
|  |  | n individuals | **27** | **70** | **30** | **20** | **100** | **120** | **2** | **20** | **40** | **3** | **11** | **80** | **120** | **50** | **593** |
| 1 | Australia, NSW | n extracts | 11 | 36 | 3 | 2 | 10 | 6 | 2 | 2 | 2 | 3 | 10 | 8 | 6 | 16 | 85 |
|  |  | n individuals | 11 | 36 | 15 | 20 | 100 | 120 | 2 | 20 | 40 | 3 | 10 | 80 | 120 | 34 | 543 |
| 2 | Australia, ACT | n extracts | 6 | 24 | 3 | - | - | - | - | - | - | - | 1 | - | - | 6 | 28 |
|  |  | n individuals | 6 | 24 | 15 | - | - | - | - | - | - | - | 1 | - | - | 6 | 40 |
| 3 | New Zealand | n extracts | 10 | 10 | **-** | **-** | **-** | **-** | **-** | **-** | **-** | **-** | **-** | **-** | **-** | 10 | 10 |
|  |  | n individuals | 10 | 10 | **-** | **-** | **-** | **-** | **-** | **-** | **-** | **-** | **-** | **-** | **-** | 10 | 10 |
| **Field populations** | | n extracts | **45** | **45** | **-** | **-** | **-** | **-** | **-** | **-** | **-** | **-** | **-** | **-** | **-** | **45** | **45** |
|  |  | n individuals | **45** | **45** | **-** | **-** | **-** | **-** | **-** | **-** | **-** | **-** | **-** | **-** | **-** | **45** | **45** |
| 1 | Australia, QLD | n extracts | 3 | 3 | - | - | - | - | - | - | - | - | - | - | - | 3 | 3 |
|  |  | n individuals | 3 | 3 | **-** | **-** | **-** | **-** | **-** | **-** | **-** | **-** | **-** | **-** | **-** | 3 | 3 |
| 2 | South Africa | n extracts | 10 | 10 | **-** | **-** | **-** | **-** | **-** | **-** | **-** | **-** | **-** | **-** | **-** | 10 | 10 |
|  |  | n individuals | 10 | 10 | **-** | **-** | **-** | **-** | **-** | **-** | **-** | **-** | **-** | **-** | **-** | 10 | 10 |
| 3 | Japan | n extracts | 6 | 6 | **-** | **-** | **-** | **-** | **-** | **-** | **-** | **-** | **-** | **-** | **-** | 6 | 6 |
|  |  | n individuals | 6 | 6 | **-** | **-** | **-** | **-** | **-** | **-** | **-** | **-** | **-** | **-** | **-** | 6 | 6 |
| 4 | Spain | n extracts | 10 | 10 | **-** | **-** | **-** | **-** | **-** | **-** | **-** | **-** | **-** | **-** | **-** | 10 | 10 |
|  |  | n individuals | 10 | 10 | **-** | **-** | **-** | **-** | **-** | **-** | **-** | **-** | **-** | **-** | **-** | 10 | 10 |
| 5 | United Kingdom | n extracts | 6 | 6 | **-** | **-** | **-** | **-** | **-** | **-** | **-** | **-** | **-** | **-** | **-** | 6 | 6 |
|  |  | n individuals | 6 | 6 | **-** | **-** | **-** | **-** | **-** | **-** | **-** | **-** | **-** | **-** | **-** | 6 | 6 |
| 6 | Chile | n extracts | 10 | 10 | **-** | **-** | **-** | **-** | **-** | **-** | **-** | **-** | **-** | **-** | **-** | 10 | 10 |
|  |  | n individuals | 10 | 10 | **-** | **-** | **-** | **-** | **-** | **-** | **-** | **-** | **-** | **-** | **-** | 10 | 10 |
| **Total** | | **n extracts** | **72** | **115** | **6** | **2** | **10** | **6** | **2** | **2** | **2** | **3** | **11** | **8** | **6** | **77** | **168** |
|  |  | **n individuals** | **72** | **115** | **30** | **20** | **100** | **120** | **2** | **20** | **40** | **3** | **11** | **80** | **120** | **95** | **638** |
