# Supplementary material for: Polyploidy versus endosymbionts in obligately thelytokous thrips
Source: BMC Evol Biol. 2015 Feb 22;15:23. doi: 10.1186/s12862-015-0304-6 (PMC4349774; doi:10.1186/s12862-015-0304-6)
Supplement: Additional file 9: Figure S1. — Mean number of female progeny of H. haemorrhoidalis (n= 10) after treatment of adult specimens (experiment 1) with different concentrations of rifampicin and tetracycline hydrochloride. Treatments did not result in reproduction of males. For 2.5% and 5% tetracycline hydrochloride, all treated mothers died after the 2nd week, and there was no offspring in the 3rd week after treatment. [file 12862_2015_304_MOESM9_ESM.doc]

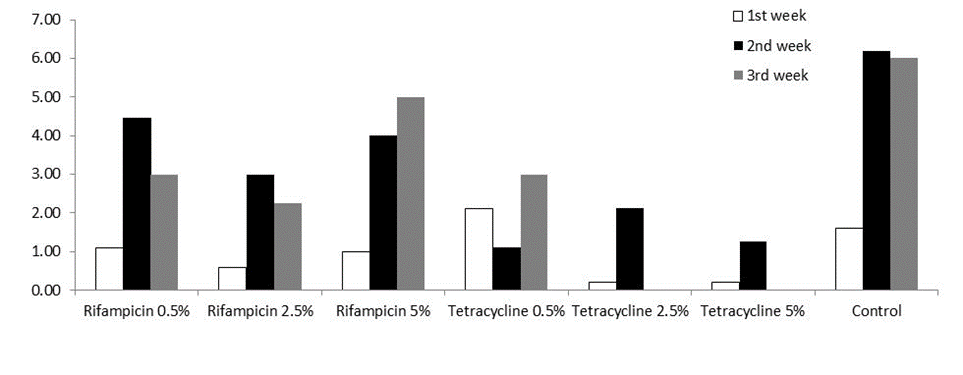


**Additional file 9:** **Figure S1.** Mean number of female progeny of *Heliothrips haemorrhoidalis* (n= 10) after treatment of adult specimens (experiment 1) with different concentrations of rifampicin and tetracycline hydrochloride. Treatments did not result in reproduction of males. For 2.5% and 5% tetracycline hydrochloride, all treated mothers died after the 2^nd^ week, so there was no offspring in the 3^rd^ week after treatment.
