# Supplementary material for: Polyploidy versus endosymbionts in obligately thelytokous thrips
Source: BMC Evol Biol. 2015 Feb 22;15:23. doi: 10.1186/s12862-015-0304-6 (PMC4349774; doi:10.1186/s12862-015-0304-6)
Supplement: Additional file 10: Figure S2. — Phylogenetic tree based on the COI gene (627bp) of H. haemorrhoidalis and other thrips species within the family of Thripidae constructed by Bayesian Inference (model GTR+G+I). Haplothrips victoriensis (suborder Tubulifera: Phlaeothripidae) was used as outgroup. Numbers at nodes represent posterior probabilities >50%. (*) One identical sequence was amplified from all 27 individuals from this study. Scale bar represents the number of nucleotide substitutions per site. [file 12862_2015_304_MOESM10_ESM.doc]

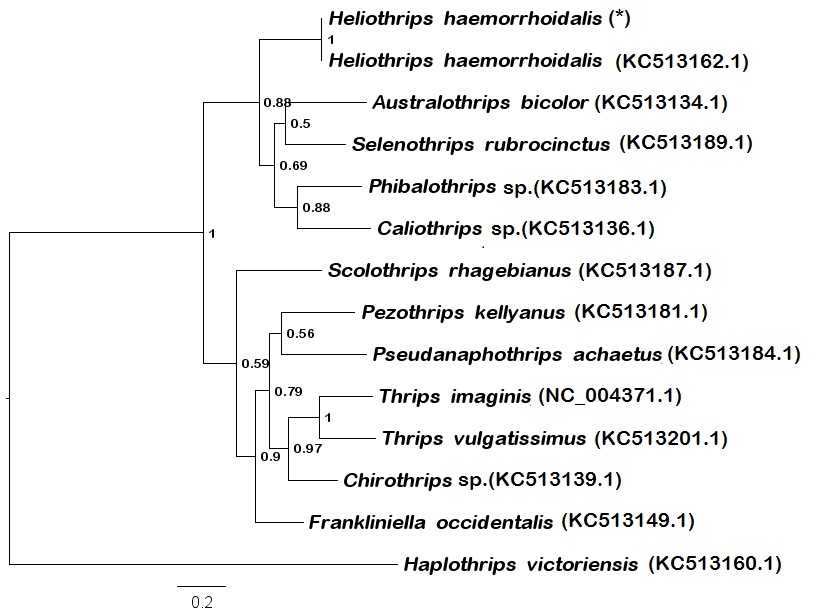


Terebrantia

Tubulifera

Panchaetothripinae

Thripinae

**Additional file 10:** **Figure S2.** Phylogenetic tree based on the *COI* gene (627bp) of *Heliothrips haemorrhoidalis* and other thrips species within the family of Thripidae constructed by Bayesian Inference (model GTR+G+I). *Haplothrips victoriensis* (suborder Tubulifera: Phlaeothripidae) was used as outgroup. Numbers at nodes represent posterior probabilities >50%. (*) One identical sequence was amplified from all 27 individuals from this study. Scale bar represents the number of nucleotide substitutions per site.
