# Supplementary material for: Polyploidy versus endosymbionts in obligately thelytokous thrips
Source: BMC Evol Biol. 2015 Feb 22;15:23. doi: 10.1186/s12862-015-0304-6 (PMC4349774; doi:10.1186/s12862-015-0304-6)
Supplement: Additional file 11: Figure S3. — Polymorphic sites within exons and introns in an H3 gene fragment (453bp) defined the different alleles of H. haemorrhoidalis. Individuals had at least two different alleles (maximum of three). The intron started with GTA (position 210-212) and ended with CAG (position 330-332). The exon of all alleles was 330bp in length. Indels are represented by a dash, while a match is represented by a point. [file 12862_2015_304_MOESM11_ESM.doc]

453bp

**Additional file 11: Figure S3.** Polymorphic sites within exons and introns in a *H3* gene fragment (453bp) defined the different alleles of *Heliothrips haemorrhoidalis.* Individuals had at least two different alleles (maximum of three) indicating that they were heterozygous. The intron started with GTA (position 210-212) and ended with CAG (position 330-332). The exon of all alleles was 330bp in length. Indels are represented by a dash, while a match is represented by a point.
