# Supplementary material for: Polyploidy versus endosymbionts in obligately thelytokous thrips
Source: BMC Evol Biol. 2015 Feb 22;15:23. doi: 10.1186/s12862-015-0304-6 (PMC4349774; doi:10.1186/s12862-015-0304-6)
Supplement: Additional file 12: Figure S4. — Polymorphic sites within exons and introns in a EF1a gene fragment (861bp) defined different alleles of H. haemorrhoidalis (allele names in the last column). Individuals had at least two alleles or a maximum of three alleles. The first intron started with GTA (position 154-56) and ended with TAG (position 224-226). The second intron started with GTA (624-626) and ended with TAG (728-730); intron 2 was variable in size; alleles A2, A3 and A9 were 83bp, while A1, A4, A5, A6, A7, A8, A11 and A12 were 85bp long; allele A10 was 101bp long. The exon sequence was 681bp long. Indels are represented by a dash, while a match is represented by a point. [file 12862_2015_304_MOESM12_ESM.doc]

861bp

**Additional file 12:** **Figure S4.** Polymorphic sites within exons and introns in a *EF1a* gene fragment (861bp) defined different alleles of *Heliothrips haemorrhoidalis* (allele names in the last column). Individuals were heterozygous (with at least two alleles or a maximum of three alleles) or homozygous. The first intron started with GTA (position 154-56) and ended with TAG (position 224-226). The second intron started with GTA (624-626) and ended with TAG (728-730); intron 2 was variable in size; alleles A2, A3 and A9 were 83bp, while A1, A4, A5, A6, A7, A8, A11 and A12 were 85bp long; allele A10 was 101bp long. The exon sequence was 681bp long. Indels are represented by a dash, while a match is represented by a point.
