# Supplementary material for: Polyploidy versus endosymbionts in obligately thelytokous thrips
Source: BMC Evol Biol. 2015 Feb 22;15:23. doi: 10.1186/s12862-015-0304-6 (PMC4349774; doi:10.1186/s12862-015-0304-6)
Supplement: Additional file 13: Figure S5. — Heliothrips haemorrhoidalis phylogenetic tree of EF1a allelic sequences (including intron) constructed with Bayesian likelihood inference (Model T92+G). Allele numbers A1-A12 are represented by collection locations. Numbers at nodes represent posterior probabilities >50%. Scale bar represents the number of nucleotide substitutions per site. [file 12862_2015_304_MOESM13_ESM.doc]

**Additional file 13:** **Figure S5.** *Heliothrips haemorrhoidalis* molecular phylogram of *EF1a* allelic sequences (including intron) constructed with Bayesian likelihood inference (Model T92+G). Allele numbers A1-A12 are represented by collection locations. Numbers at nodes represent posterior probabilities >50%. Scale bar represents the number of nucleotide substitutions per site.
